# Mechanical, Viscoelastic, Thermal and Morphological Properties of Hexagonal Boron Nitride (h-BN)-Doped Polyester Nano-Gelcoat Under Hydrothermal Aging

**DOI:** 10.3390/polym18060743

**Published:** 2026-03-18

**Authors:** Gokhan Demircan, Mustafa Ozen, Cennet Cakmak, Busra Nur Celik, Abuzer Acikgoz, Murat Kisa

**Affiliations:** Mechanical Engineering Department, Engineering Faculty, Harran University, Sanliurfa 63000, Türkiye; mustafaozen@harran.edu.tr (M.O.); cnntcakmak7@gmail.com (C.C.); busranurcelik26@hotmail.com (B.N.C.); a.abuzer@harran.edu.tr (A.A.); mkisa@harran.edu.tr (M.K.)

**Keywords:** nano-gelcoat, hexagonal boron nitride (h-BN), mechanical properties, hydrothermal aging

## Abstract

Fiber-reinforced polymer (FRP) composites used in marine environments suffer progressive degradation due to hydrothermal aging, which undermines their structural, physical and morphological integrity. In this study, a novel polyester-based nano-gelcoat reinforced with hexagonal boron nitride (h-BN) nanoparticles was developed as an advanced FRP composite coating for marine applications. Glass fiber/epoxy laminates coated with h-BN/polyester nano-gelcoat were subjected to accelerated hydrothermal aging (immersion in 80 °C artificial seawater for 90 days). Mechanical (tensile/flexural tests), viscoelastic (creep and stress relaxation), thermal (DSC/TGA), and morphological (optical microscopy/SEM) analyses were performed on aged and unaged samples. The h-BN-enhanced nano-gelcoat increased the composite’s resistance to hydrothermal aging. In particular, the optimally doped nano-gelcoat (~1 wt% h-BN) retained the highest tensile and flexural strength and modulus, reducing the property losses seen in the unreinforced system by about half (flexural strength 531.29 MPa vs. 1070.52 MPa for the uncoated laminate). Thermal analysis indicated elevated decomposition onset temperatures and higher char yields with h-BN, confirming improved thermal stability. Morphological observations revealed well-dispersed h-BN at 1 wt% with minimal microcracking, whereas higher filler loadings led to agglomeration. Additionally, a TOPSIS-based multi-criteria decision-making (MCDM) analysis was performed across mechanical, viscoelastic, and thermal metrics, which identified the 1 wt% h-BN coating as the most balanced formulation after hydrothermal aging.

## 1. Introduction

Gelcoats constitute the primary exterior layer in the construction of fiber-reinforced polymer (FRP) marine hulls, applied to the mold before laminate lay-up and curing to deliver a smooth cosmetic finish while acting as the first line of environmental protection [1,2]. This highly cross-linked layer is typically produced from polyester or vinyl ester resins. It reduces water vapor permeability, which limits water ingress and helps prevent osmotic blistering. At the same time, it shields the underlying fiber–matrix architecture from ultraviolet (UV) radiation, weathering, and chemical attack [3,4]. Under service conditions, prolonged immersion at elevated temperatures accelerates hydrothermal aging. Moisture uptake, plasticization, interfacial degradation, and surface roughening progressively develop in the material. These effects, in turn, decrease its mechanical and viscoelastic performance, thermal stability, and morphological integrity [5,6]. To address these limitations, recent research has focused on multifunctional nanostructured gelcoats with enhanced barrier and heat-dissipation characteristics [7].

Nano hexagonal boron nitride (h-BN) is a potential ceramic material for fabricating nanostructured materials [8]. It is a chemically inert, thermally conductive, platelet-structured ceramic and is a promising dopant capable of extending diffusive pathways and distributing thermal loads, thereby improving resistance to hydrothermal aging [9,10]. In polyester matrices, h-BN can (i) elongate diffusive pathways and reduce effective water permeability via a tortuous-path barrier effect, (ii) dissipate thermally induced stresses near the surface owing to in-plane heat conduction, and (iii) constrain chain mobility to moderate plasticization and hydrolytic scission under hydrothermal aging [11,12]. Using electrochemical impedance spectroscopy, h-BN/polymer hybrids applied on stainless steel were shown to markedly reduce corrosion in simulated seawater [13]. Based on these experiments, a later review explained exactly how h-BN systems protect against corrosion. It highlighted three main factors: complex pathways that block moisture, increased water repellency, and electrical insulation that stops unwanted galvanic reactions [14]. In parallel, broader design principles were established for using boron nitride nanosheets (BNNSs) in polymer anticorrosion coatings [15]. These principles cover their intrinsic properties, scalable top-down and bottom-up synthesis routes, and dispersion/interfacial engineering strategies that together enhance barrier performance. To turn these ideas into an active, self-protecting system, researchers embedded special h-BN nanocontainers (mBN@BTA) into an epoxy base. This combination gives steel a dual defense: a physical shield to block moisture and an active chemical treatment to stop rust [16]. Extending the use of h-BN to electrical insulation applications, silane-modified h-BN (3-glycidoxypropyltrimethoxysilane) was dispersed into unsaturated polyester (1–10 wt%) to form insulation-varnish composites [17].

Recent studies identify hexagonal boron nitride nanosheets (h-BN/BNNSs) as electrically insulating and chemically stable barrier fillers. These fillers can improve corrosion, oxidation, and wear resistance in polymer coatings. However, most of this literature focuses on metallic substrates such as copper, carbon steel, and stainless steel [18,19,20,21]. In these systems, coating performance is generally evaluated in terms of electrochemical corrosion and oxidation protection. In contrast, FRP gelcoat or top-coat systems are polymer-based surface layers applied to composite substrates. Therefore, their function is not limited to blocking water and salt ingress. They must also maintain coating/substrate compatibility and resist hydrothermal degradation phenomena such as plasticization, hydrolysis, swelling, blistering, and the resulting thermo-mechanical deterioration [22]. Although a limited number of studies have examined nano-gelcoats for marine FRP applications, these studies have mainly focused on nanoclay- or TiO_2_-based systems and water-absorption behavior. As a result, h-BN-containing FRP gelcoat systems under hydrothermal aging remain largely unexplored [23]. Accordingly, the present study addresses a more specific gap. It investigates the use of h-BN in a polyester/PDMS nano-gelcoat for GFRP. The main focus is on the retained mechanical, viscoelastic, thermal, and morphological performance after severe hydrothermal seawater exposure.

Polydimethylsiloxane (PDMS) was also used to improve the hydrophobicity of the nano-gelcoat in this study. It is widely used as a functional silicone additive in coating formulations. At low dosages (from tens–hundreds of ppm up to a few wt%), it lowers surface tension, improves substrate wetting and flow/leveling, and helps defoaming/degassing. These effects arise mainly from physical interactions, as unmodified PDMS oils lack reactive groups [24,25,26]. In cured networks, PDMS at or near the surface also imparts durable hydrophobicity, which can reduce moisture uptake at the coating/air interface. Taken together, these gaps and opportunities motivate the present study. We examine h-BN-modified polyester nano-gelcoats, formulated with PDMS as a processing and surface energy modifier, applied directly to FRP surfaces. In particular, we explicitly track viscoelastic property retention under marine-relevant conditioning [27].

In this study, an h-BN/PDMS-modified polyester nano-gelcoat was applied directly to FRP laminates, and its durability was evaluated under accelerated hydrothermal aging (80 °C artificial seawater for 90 days). Mechanical performance and viscoelastic behavior were examined from FRP laminates, and nano-gelcoats were investigated with morphological observations and thermal responses.

## 2. Materials and Methods

### 2.1. Materials

Woven glass fiber/epoxy plates (GFRPs) were manufactured by vacuum-assisted resin infusion molding (VARIM) and used as structural substrates. The epoxy system consisted of LR1160 resin and LH1160 hardener (Propox, Dost Kimya, Istanbul, Türkiye) mixed at 4:1 by mass. Lay-ups employed nine glass fabric plies. Plates were produced to an average thickness of 2.5 ± 0.1 mm.

The nano-gelcoat matrix was an unsaturated polyester resin (CE 92 N8, Camelyaf Resins, Istanbul, Türkiye), diluted with styrene monomer at 10 wt% of the polyester to attain sprayable viscosity. Polydimethylsiloxane (PDMS, Sigma-Aldrich, St. Louis, MO, USA) was used at 2 wt%. Cobalt octoate and methyl ethyl ketone peroxide were used at 0.18 wt% and 1.5 wt%, respectively, relative to the polyester. Hexagonal boron nitride (h-BN) nanopowder (99.85+%, Size: 65–75 nm, Nanografi, Ankara, Türkiye) was incorporated at 0–5 wt% relative to polyester. The coating was applied onto the GFRP substrates as a three-pass spray.

### 2.2. Production of Composite Plates and Coating Process

Glass fiber/epoxy composite plates were produced by vacuum-assisted resin infusion molding (VARIM) [28]. The lay-up stack was prepared to approximately 345 mm × 465 mm using release film, peel-ply, infusion mesh, spiral infusion lines, and vacuum bag. All vacuum lines were leak-checked prior to infusion. The epoxy system (LR1160 resin/LH1160 hardener) was mixed at 4:1 by mass, mechanically stirred, and degassed for ~15 min in a vacuum chamber to minimize entrapped air. With the vacuum pump running, resin was drawn through the preform until complete wet-out was achieved. The vacuum was turned off, and the laminate was cured at 80 °C for 15 h. Plates were then removed from consumables and given a post-cure on a heated bench for an additional 15 h.

**Figure 1:**
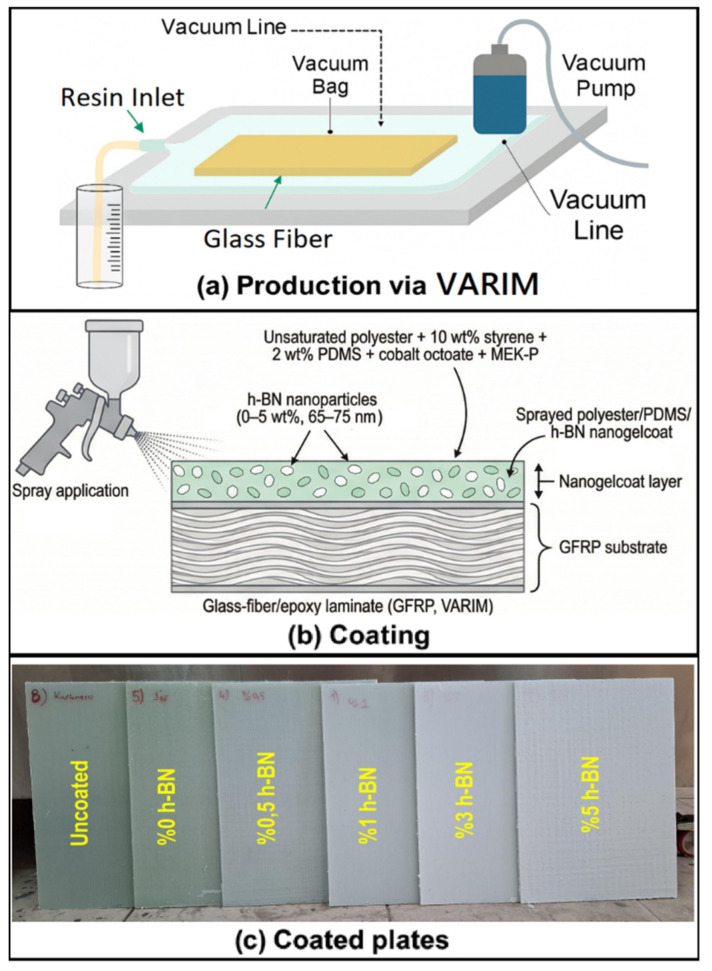
Production of composite plates and coating process.

Prior to coating, laminate surfaces were cleaned of all processing residues and inspected to ensure a uniform, defect-free substrate suitable for spray application. Nano-gelcoat batches were prepared by diluting unsaturated polyester (CE 92 N8) with 10 wt% styrene to attain sprayable viscosity, adding PDMS at 2 wt%, cobalt octoate at 0.18 wt% as accelerator, and MEK-P at 1.5 wt% as initiator (all percentages relative to polyester). For h-BN-filled formulations (0–5 wt% h-BN), the prescribed nanopowder mass was dispersed into the polyester–styrene blend by probe ultrasonication in an ice bath (2 h total at ~70% amplitude, 2 s on/3 s off duty cycle) while magnetically stirred (first hour 400 rpm, second hour 500 rpm). PDMS and cobalt octoate were then incorporated. Throughout sonication the beaker was kept in an ice bath to limit cavitation heating. Immediately before each spray pass, MEK-P was added and mixed ~2 min. Coatings were applied to GFRPs. All production and coating stages are represented in Figure 1. To improve reproducibility, all nano-gelcoat formulations were applied to cleaned, defect-free GFRP substrates using the same three-pass spray procedure. Before application, the resin system was adjusted to a sprayable viscosity with 10 wt% styrene. Coating thickness was measured at multiple locations. These measurements were also supported by cross-sectional SEM observations. The results showed a coating thickness of approximately 400–450 µm. This confirmed reasonable uniformity among the sprayed layers.

### 2.3. Artificial Aging Process

**Figure 2:**
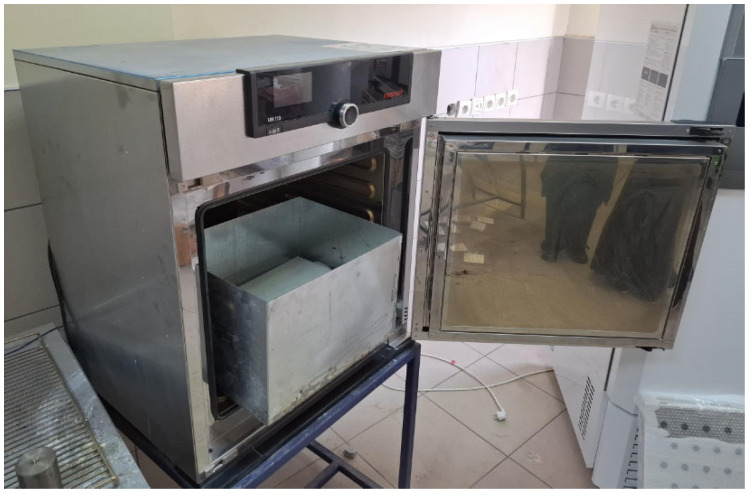
Hydrothermal cabinet.

Coated and uncoated specimens were loaded according to a fixed rack arrangement, and the aging programs were initiated. Specimens were immersed in an artificial seawater (3.5 wt% NaCl) bath prepared by adding 3.5 wt% rock salt to tap water and held at 80 °C for 90 days in a hydrothermal cabinet/furnace [29]. Bath concentration and temperature were maintained continuously for the duration of exposure. The hydrothermal cabinet is shown in Figure 2. Although 80 °C is higher than normal seawater temperatures, it was used to accelerate hydrothermal aging because it is close to the Tg range of the polyester-based coatings, where degradation effects become more noticeable [30]. The 90-day aging period was selected because it is commonly used in the literature and is long enough to cause measurable damage within a practical laboratory time [31].

### 2.4. Tests and Characterization

Standard tensile and three-point bending tests were performed following ASTM D3039 [32] and ASTM D790 [33], respectively. Time-dependent behavior was quantified by creep and stress relaxation protocols. Creep was conducted for 3 h at 40% of the maximum tensile stress, while stress relaxation was recorded for 3 h at 40% of the maximum tensile strain obtained from the tensile test. Mechanical test standards were given in Table 1.

Microstructural features and matrix–fiber/interface changes were examined by SEM (Zeiss Evo 50, Carl Zeiss SMT Ltd., Cambridge, UK). Degradation and thermal transitions were evaluated by TGA/DTA using an SII EXSTAR TG/DTA 7300 (SII NanoTechnology Inc., Chiba, Japan) system. The measurements were performed under a nitrogen atmosphere at a flow rate of 100 mL/min. The heating rate was 10 °C/min, and the temperature range was 30–600 °C. DSC analysis was carried out using a PerkinElmer DSC 8000 instrument (PerkinElmer, Inc., Waltham, MA, USA). Approximately 6–7 mg of sample was placed in sealed aluminum pans. The measurements were also conducted under nitrogen at 100 mL/min. A heating rate of 10 °C/min was applied over the temperature range of 20–600 °C. Enthalpy changes and melting behavior were monitored using an empty pan as the reference.

### 2.5. Multi-Criteria Decision-Making Method (MCDM)

The Technique for Order Preference by Similarity to Ideal Solution (TOPSIS) was employed as the multi-criteria decision-making (MCDM) process. A set of m alternatives, each representing a distinct h-BN content, was evaluated against *n* criteria grouped into three categories: (i) mechanical: tensile strength, tensile modulus, flexural strength, flexural modulus; (ii) viscoelastic: quasi-steady creep rate, ΔJ, ΔEc, stress retention; and (iii) thermal: T_onset_, T_max_, T_g_. Criterion weights satisfied the unit-sum constraint (Equation (1)) and were defined under four scenarios: Case-1 (equal weights, %33.33, %33.33, %33.33); Case-2 (%60, %20, %20); Case-3 (%20, %60, %20); and Case-4 (%20, %20, %60) with category shares for mechanical/viscoelastic/thermal.

(1) $$\sum_{j=1}^{n}w_{j}=1$$

Category shares were uniformly distributed among criteria within each category (Equation (2)). Criteria and case conditions are summarized in Table 2.

(2) $$w_{j}=\frac{\alpha_{c}}{n_{c}}\;\mathrm{for}\;\mathrm{any}\;\mathrm{criterion}\;j\;\mathrm{in}\;\mathrm{category}\;c\in \left\{\mathrm{Mechanical},\;\mathrm{Viscoelastic},\;\mathrm{Thermal}\right\}$$

The computational steps followed the standard TOPSIS pipeline, beginning with vector normalization (Equation (3)) and the formation of the weighted normalized matrix to identify the ideal best and worst values. Subsequently, we computed the Euclidean separation measures from these ideals (Equations (4) and (5)) and evaluated the final closeness coefficient (Equation (6)).

(3) $$r_{ij}=\frac{x_{ij}}{\sqrt{\sum_{i=1}^{m}x_{ij}^{2}}}$$

(4) $$S_{i}^{*}=\sqrt{\sum_{j=1}^{n}(v_{ij}-v_{j}^{*}{)}^{2}}$$

(5) $$S_{i}^{-}=\sqrt{\sum_{j=1}^{n}(v_{ij}-v_{j}^{-}{)}^{2}}$$

(6) $$C_{i}^{*}=\frac{S_{i}^{-}}{S_{i}^{*}+S_{i}^{-}}$$

**Figure 3:**
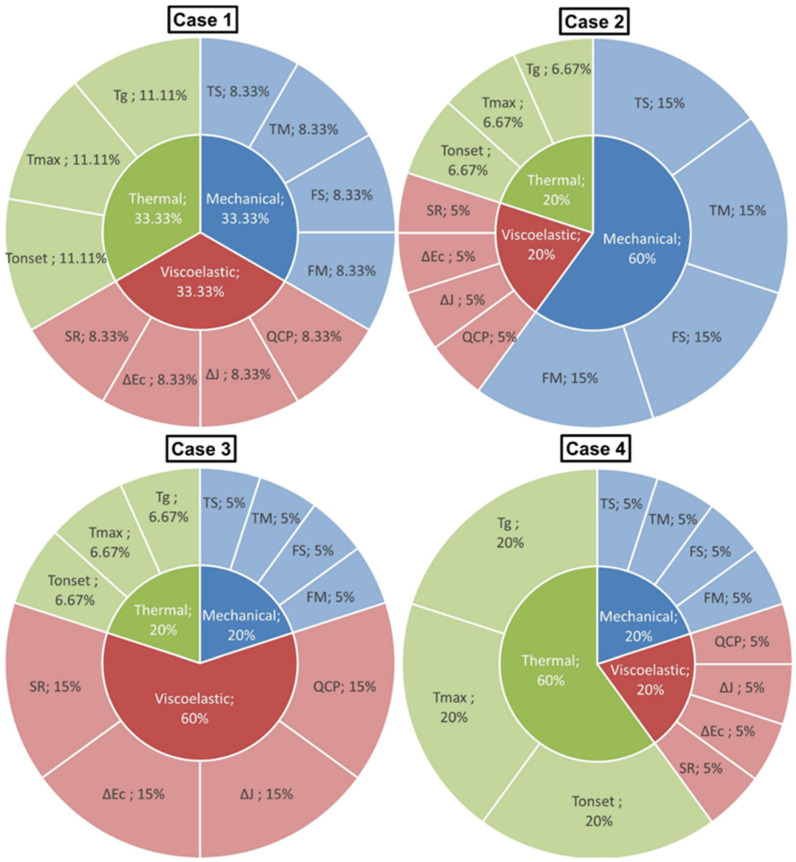
Pie charts of case conditions for MCDM.

Alternatives were ranked in descending order of the closeness coefficient for each scenario. Criterion types were assigned on physical grounds: strengths, modulus, stress retention, T_onset_, T_max_, and T_g_ were treated as benefit criteria, whereas the creep rate, ΔJ, and ΔE_c_ were treated as cost criteria. All steps were implemented in Microsoft Excel using explicit formulae. All case conditions were shown in Figure 3.

## 3. Results and Discussions

### 3.1. Mechanical Tests

#### 3.1.1. Tensile and Flexural Test Results

**Figure 4:**
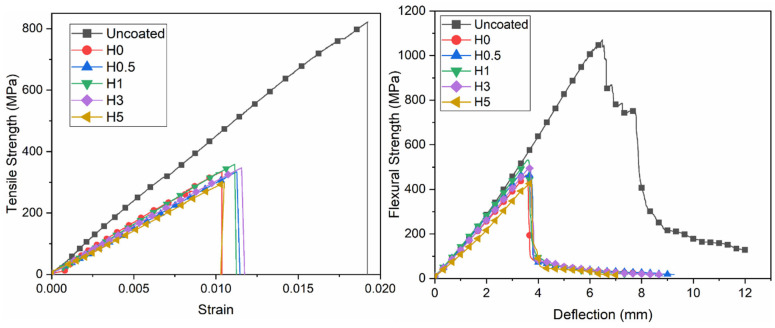
Tensile and flexural test curves.

The tensile and flexural test results for the hydrothermally aged composites are summarized in Table 3. The corresponding strength–strain and strength–deflection curves are shown in Figure 4. For easier interpretation of the mechanical degradation after aging, the percentage loss values of tensile strength (TS), modulus of elasticity (MOE), flexural strength (FS), and flexural modulus (FM) were additionally calculated relative to the uncoated control, as presented in Table 3. Among the aged groups, H1 showed the lowest losses in TS (56.4%), MOE (27.9%), FS (50.4%), and FM (10.0%), indicating the best overall mechanical property retention under the applied hydrothermal aging condition.

Among all groups, the uncoated composite exhibited the highest tensile performance, with a maximum tensile strength of 822.04 MPa and an elastic modulus of 46.09 GPa, reflecting the as-fabricated laminate’s maximum load-bearing capacity through efficient fiber–matrix load transfer. In contrast, all hydrothermally aged, coated specimens showed pronounced degradation; for the 0 wt% h-BN coating (H0), the tensile strength and modulus decreased to 332.08 MPa and 31.90 GPa, respectively. The most favorable response within the hydrothermal series was observed for the 1 wt% h-BN specimen (H1), with a tensile strength of 358.75 MPa and an elastic modulus of 33.22 GPa. Increasing the h-BN content to 3 wt% (H3) and 5 wt% (H5) led to further losses, most notably in H5 (298.77 MPa; 29.07 GPa). This degradation is consistent with nanoparticle agglomeration at higher loadings, which disrupts coating continuity and weakens the interphase. Hydrothermal exposure also promoted coating crack formation and fragmentation, ultimately compromising the barrier function. It is known that the addition of BN nanoparticles in epoxy-based systems improves the preservation of mechanical properties due to aging [36]. A previous study under hydrothermal conditions reported that epoxy modified with h-BN or h-B4C showed a 16% drop in storage modulus and an 18% drop in tensile strength after 60 days [37].

Hydrothermal aging resulted in pronounced reductions in both flexural strength and modulus. The uncoated laminate provided the highest baseline performance. Among the coated and aged specimens, the best performance was obtained at 1 wt% h-BN (H1), with a flexural strength of 531.29 MPa (approximately a 50% decrease relative to the uncoated baseline) and a flexural modulus of 34.81 GPa (≈10% reduction), indicating that 1 wt% is near-optimal for property retention. At the highest loading (H5, 5 wt%), the strength and modulus fell to 430.19 MPa and 26.91 GPa (≈60% and ≈30% reductions), consistent with h-BN agglomeration at elevated loadings that disrupts coating continuity and weakens the interphase. Incorporating h-BN into unsaturated polyester resins increases the thermal stability and hydrophobicity of the composite, leading to increased durability under hydrothermal conditions [17].

#### 3.1.2. Creep Test Results

**Figure 5:**
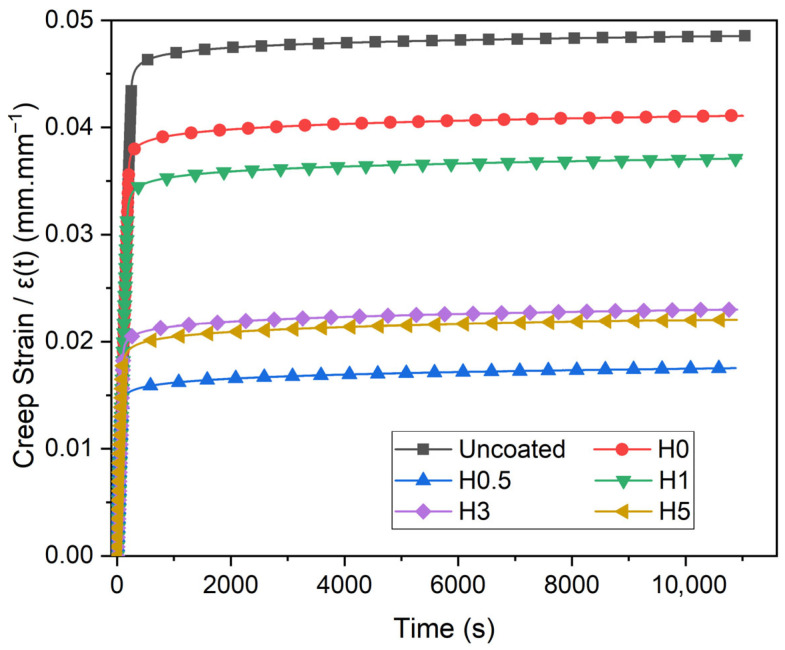
Creep strain–time responses.

**Figure 6:**
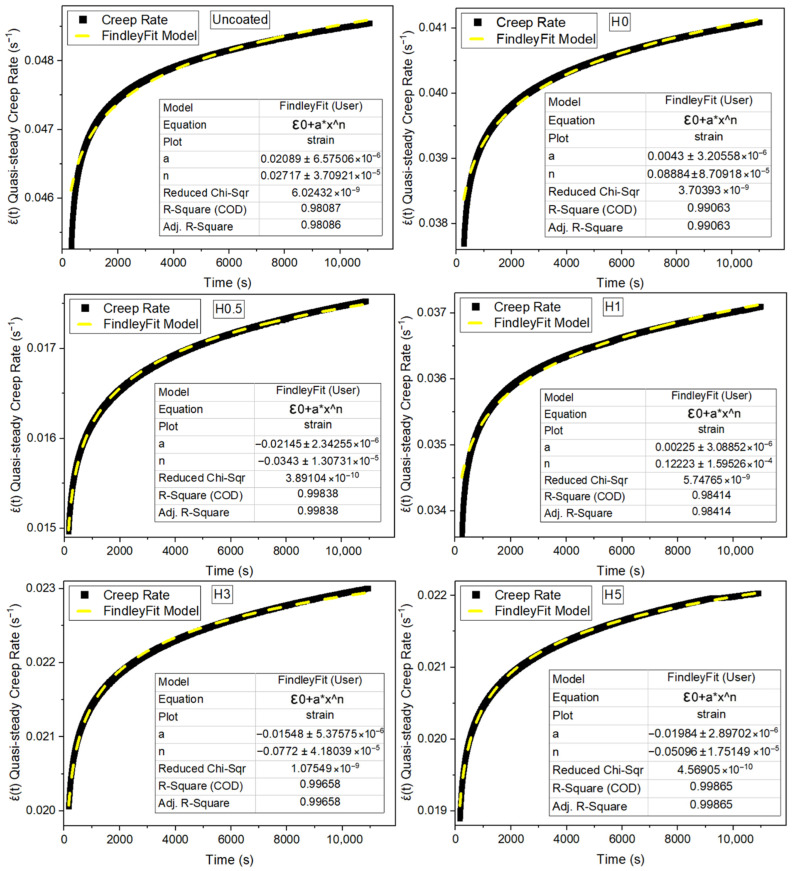
Findley-fit creep rate curves (* denotes multiplication).

The numerical results of the creep tests are shown in Table 4. The corresponding creep strain–time responses are plotted in Figure 5, and the Findley-fit creep rate curves are presented in Figure 6. $E_{cp}\left(t\right)$ denotes the creep modulus in the plateau (quasi-steady) regime, whereas $E_{c3h}\left(t\right)$ denotes the creep modulus evaluated at t = 3 h.

The Findley model is a two-parameter power law commonly used to describe primary creep of polymers and FRP composites under a constant uniaxial stress within the linear viscoelastic (LVE) range. Findley power law model is presented in Equation (7).

(7) $$\varepsilon \left(t\right)=\varepsilon_{0}+At^{n}$$

Here, ε(t) is the creep strain at time $t;\varepsilon_{0}$ is the instantaneous elastic strain; A is the amplitude of the time-dependent creep strain; and 0<n<1 is the creep exponent that governs the curvature (the creep rate follows from Equation (7) as $\dot{\varepsilon }(t)=Ant^{n-1}$). For explicit stress dependence, the model can be written in compliance form in Equation (8);

(8) $$J\left(t\right)=J_{0}+Ct^{n}$$

so that under a constant stress $\sigma_{0}$ we have $\varepsilon (t)=\sigma_{0}J(t)$, with the identities $\varepsilon_{0}=\sigma_{0}\;J_{0}=\sigma_{0}/E_{0}$ and $A=\sigma_{0}C\left(E_{0}=1/J_{0}\right)$; the apparent creep modulus then reads $E_{c}(t)=\sigma_{0}/\varepsilon (t)=1/J(t)$.

All creep curves in this study remained in the primary regime throughout the 3 h hold. No transition to secondary (steady state) or tertiary creep was observed. Consequently, the Findley power law in Equation (7) was fitted only to the primary creep portion of each record. Specifically, once the axial stress reached a stable plateau at time $t_{0}$, we excluded the first 60 s to remove short transients associated with stress ramping and machine/compliance settling and performed the regression over the interval $\left[t_{0}+60\;s,\;t_{\mathrm{end}}\right]$. This choice is standard for FRP laminates tested at subcritical stress levels (where a steady-state creep rate typically does not develop over laboratory time scales) and ensures that the reported $J_{0}$, a, and n parameters quantify the primary creep response of the coated and uncoated laminates.

In Table 4 relative to the uncoated sample, the H-series shows consistently higher quasi-steady creep rates ranging from 4.12844 × 10^−7^ s^−1^ in H1 (+5.8%) to 6.19735 × 10^−7^ s^−1^ in H5 (+58.8%). The same series exhibits markedly larger creep-compliance increases, ΔJ, spanning 13.78% (H0, +35.4%) to 26.98% (H5, +165.0%). The increase in creep strain, Δε, tightens around the baseline (H0.5, −23.6%; (H0, +0.4%). Modulus responses diverge with the plateau creep modulus E_cp_ (H1, −33.3%; H0.5, +31.5%), while E_c3h_ ranges from 3.84 GPa (H1, −36.0%) to 7.00 GPa (H0.5, +16.7%). The change in creep modulus ΔE_c_ increases across all aged groups, H0.5 yields the lowest strain increase and the highest modulus (both plateau and 3h), H1 achieves the lowest $\dot{\varepsilon }$ among aged samples but with the lowest modulus, and H5 shows the most severe deterioration, combining the highest $\dot{\varepsilon }$, ΔJ, and ΔE_c_.

The creep data are consistent with two well-established mechanisms: (i) water-induced plasticization of the epoxy matrix, which decreases the glass transition temperature and accelerates viscoelastic creep [38], and (ii) interfacial weakening that facilitates micromechanical shear sliding along the fiber/matrix interface [39]. Accordingly, all aged groups exhibit increased creep compliance (ΔJ%) and higher quasi-steady creep rates, together with larger losses in the 3 h creep modulus (ΔE_c_%). Incorporation of 1 wt% h-BN gives the most pronounced short-term improvement among the aged coatings. It yields the lowest quasi-steady creep rate, and the smallest modulus drop. This behavior is attributed to h-BN creating tortuous diffusion paths that slow water ingress and, when well dispersed, strengthening the matrix/interphase region [40]. At higher loadings (H3, H5), however, the creep response deteriorates again, in line with widely reported agglomeration and wettability/interfacial adhesion issues that disrupt stress transfer [41]. The uncoated specimen serves as the most stable short-term reference, whereas H0 displays the canonical hydrothermal-aging signature with marked increases in ΔJ% and creep rate. Within the aged series, the H1 formulation most effectively suppresses creep, confirming it as the optimum nano-filler dosage. Conversely, the performance drop in H3 and H5 indicates poor particle dispersion at higher filler levels, while the inconsistent behavior of H0.5 likely stems from uneven particle distribution.

#### 3.1.3. Stress Relaxation Test Results

**Figure 7:**
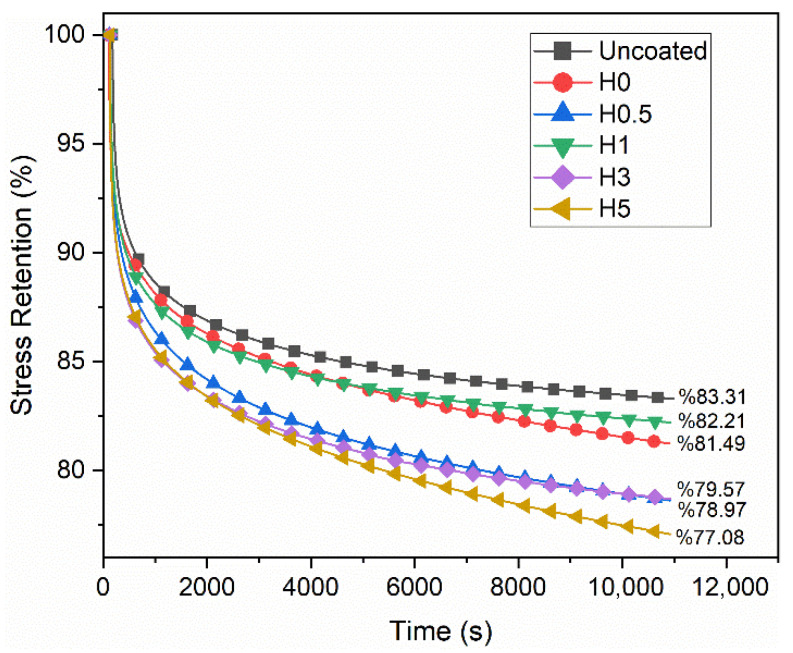
Stress retention curves.

**Figure 8:**
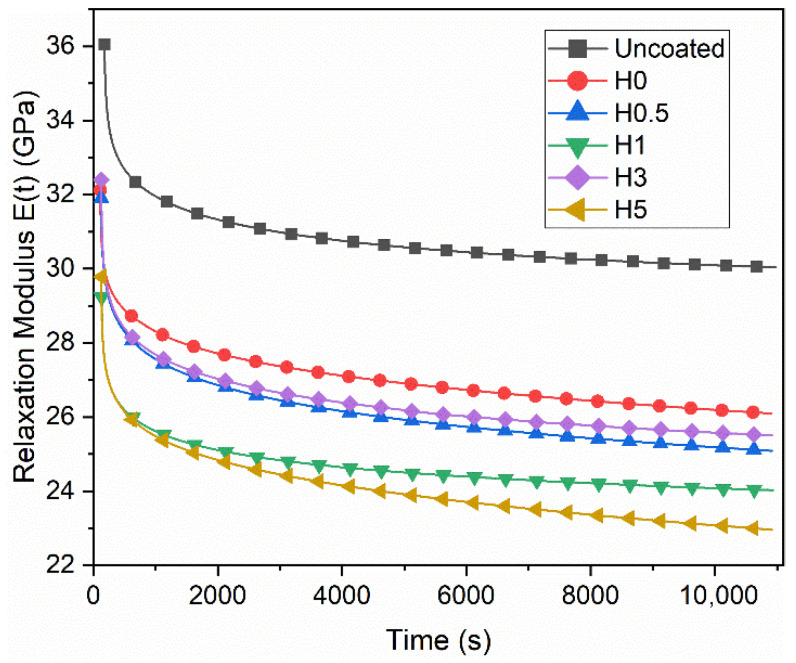
Change in relaxation modulus.

The numerical stress relaxation results are shown in Table 5. The corresponding stress retention curves are shown in Figure 7, and the changes in the relaxation modulus are plotted in Figure 8. Here, $E_{rp}(t)$ denotes the relaxation modulus in the quasi-steady (plateau) regime, whereas $E_{r3h}(t)$ denotes the relaxation modulus evaluated at t=3 h.

Relative to the uncoated reference, all coated groups exhibit a pronounced decrease in the relaxation modulus $E_{r}$ and a lower stress retention ratio. In the uncoated specimen, the plateau relaxation modulus is $E_{rp}=36.05\;\mathrm{GPa}$ and the 3 h modulus is $E_{r3h}=30.04\;\mathrm{GPa}$, corresponding to a stress retention of 83.31%. Consistent with literature expectations, seawater plasticizes the epoxy matrix, which lowers the $T_{g}$ and accelerates viscoelastic chain mobility. This relaxation process is further amplified by micro-damage and interfacial debonding, ultimately leading to a decrease in both $E_{r}$ and the $E_{r3h}/E_{rp}$ ratio [42,43].

With respect to h-BN content, the highest stress retention among the aged samples is achieved at 1 wt% (H1, 82.21%), whereas the lowest is observed at 5 wt% (H5, 77.08%). This behavior suggests that a small, well-dispersed amount of h-BN effectively limits water uptake by acting as a diffusion barrier. Furthermore, the material’s high thermal conductivity minimizes local stress concentrations and subsequent interfacial damage. At higher loadings (≥3–5 wt%), however, agglomeration, viscosity-related processing defects, and poor wetting increase voids and defects, which in turn accelerate relaxation [44,45]. Because absolute $E_{r}$ levels can vary across groups due to differing $0.40\;\varepsilon_{max}$ setpoints and potential nonlinearities (e.g., coating cracking captured by the extensometer), ratio-based metrics are stronger for comparisons. Here, stress retention equals $E_{r3h}/E_{rp}$, which numerically complements the short-term creep metric $\Delta E_{c}\%\approx 100\;(1-E_{r3h}/E_{rp})$. The fact that H1 also ranks best in tensile/flexural retention while H5 performs worst across both mechanical and relaxation metrics indicates strong internal consistency in the dataset. In summary, hydrothermal seawater exposure accelerates viscoelastic relaxation and depresses 3 h stress retention, with 1 wt% h-BN emerging as the most effective formulation for limiting degradation.

### 3.2. Thermal Tests

In this study, thermal characterization was deliberately performed on the standalone coatings (nano-gelcoat), whereas mechanical testing was carried out on the coated FRP laminates. This design choice separates the material’s own properties from the effects of the overall system. This prevents the coating’s thermal signature from getting mixed up with the heavy and complex base material. Differential methods such as DSC and TGA are highly sensitive to sample heat capacity, mass fraction, and homogeneity. When a thin coating is bonded to a thick, fiber-rich laminate, the laminate dominates the heat flow. As a result, the coating’s glass transition may become difficult to detect. The laminate can also mask the coating’s relaxation enthalpies and degradation onsets. This situation also introduces baseline artifacts, which make coating-specific parameters difficult to identify and reproduce. By testing free coatings, we quantify formulation-level thermal stability (e.g., T_g_, decomposition temperatures, char yield) attributable solely to the coating stability and fillers, enabling fair comparisons across compositions and aging histories. In contrast, the main structural loads in service are carried by the laminate, not the coating. The coating mainly works at the interface as a barrier, adhesion promoter, and defect shield. Therefore, its mechanical importance should be evaluated at the component scale, where coating/substrate interactions, stress transfer, and aging-barrier effects appear in measurable tensile and flexural responses. This complementary approach maximizes the reliability of the coating’s thermal metrics. At the same time, it preserves the practical relevance of the coated structure’s mechanical performance and provides a clear structure–property–performance link, without mixing substrate-driven thermal artifacts with true coating effects [46].

#### 3.2.1. Thermogravimetric Analysis (TGA) Test Results

**Figure 9:**
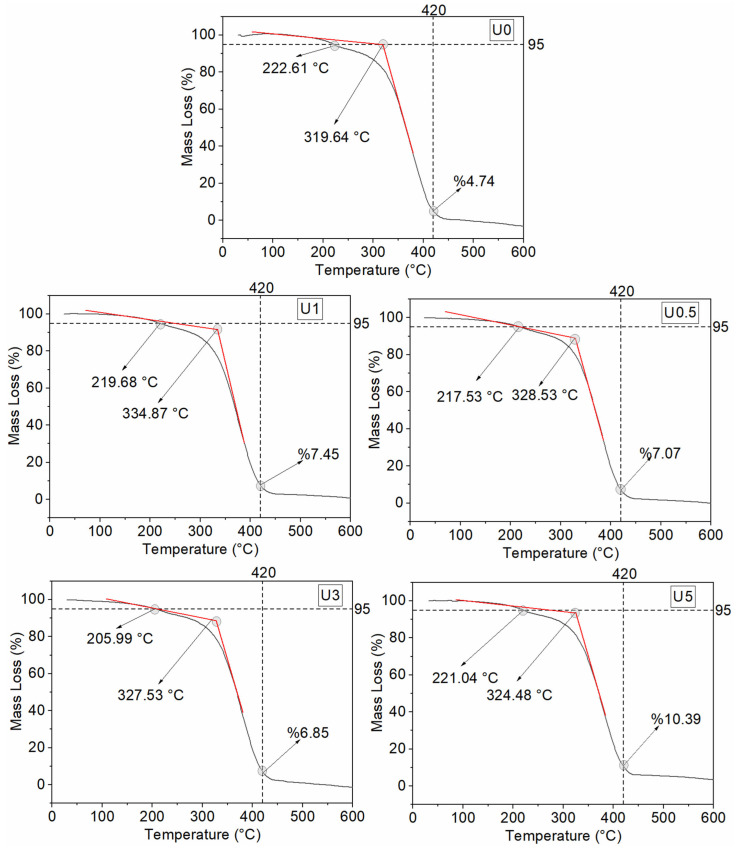
TGA curves of unaged coatings.

**Figure 10:**
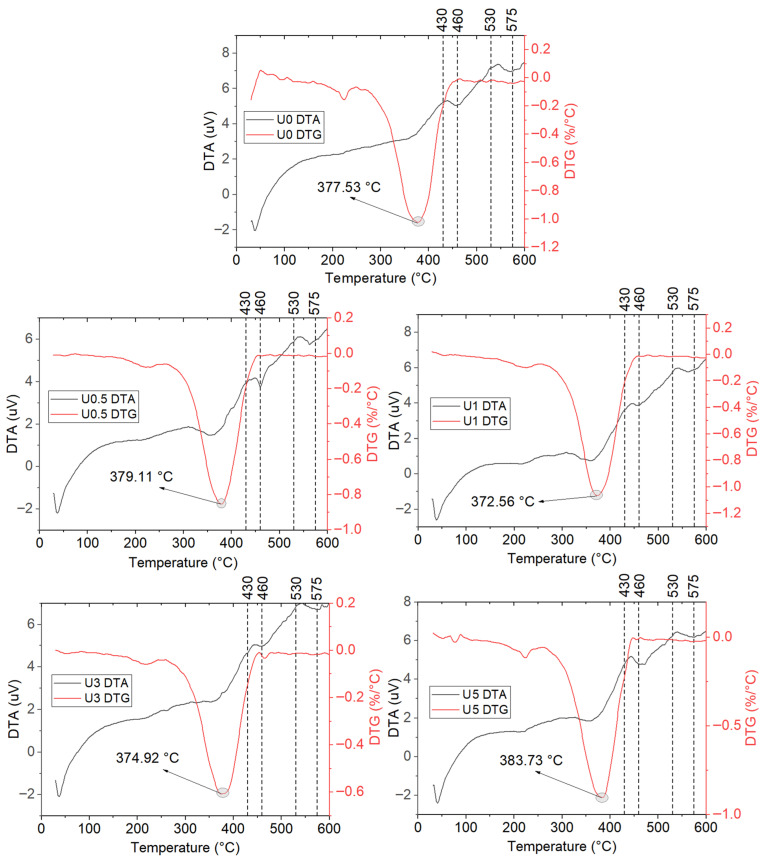
DTA and DTG curves of Unaged Coatings.

The coatings (nano-gelcoat) were classified into two series: unaged (U) and hydrothermally aged (H). The TGA results for the unaged specimens are reported in Table 6. The corresponding TGA mass-loss curves are presented in Figure 9, and the DTA and DTG curves are shown in Figure 10. In the TGA tables, $T_{5}$ denotes the temperature at 5% mass loss, $T_{\mathrm{onset}}$ is the onset decomposition temperature, $T_{max}$ is the temperature at the maximum degradation rate, and $W_{\mathrm{res},420}$ and $W_{\mathrm{res},600}$ are the residual mass at 420 °C and 600 °C, respectively.

In the unaged coatings, the TGA results reveal two distinct thermal benefits. At low h-BN loadings, the decomposition onset is shifted to higher temperatures, whereas at high loadings the main degradation is delayed. Specifically, the h-BN-free specimen (U0) exhibited a $T_{onset}$ value of 319.64 °C, while the addition of 0.5 and 1 wt% h-BN increased $T_{onset}$ markedly to 328.53 and 334.87 °C, respectively. This behavior indicates that the barrier effect of h-BN restricts heat and volatile diffusion [41]. By contrast, in U3, T5 decreases to 206.0 °C, suggesting that early mass loss is dominated by residual styrene, volatile PDMS cyclics, and possible catalytic effects of accelerator/hardener remnants [47].

With respect to the maximum degradation rate, $T_{max}$, the trend differs: a slight increase is observed at 0.5 wt% (379.11 °C), while 5 wt% h-BN yields the highest value in the series ($T_{max}=383.7\;{}^{\circ}C)$. This indicates that at higher filler contents the “heat-sink” effect of h-BN becomes dominant. Input heat does not localize but is rapidly redistributed owing to the high thermal conductivity and thermal mass, thus shifting the peak degradation to higher temperatures [48]. The residual mass at 420 °C rises approximately linearly with h-BN content from 4.74% in U0 to 10.39% in U5, consistent with thermally inert inorganic residue and a barrier-induced extension of diffusion pathways that slows mass loss at intermediate temperatures [49]. The ≈0% residue observed at 600 °C for all samples underscores the sensitivity of the endpoint to the test atmosphere and flow/calibration settings.

From an application standpoint, the best initial thermal stability is obtained at 1 wt% h-BN. This composition most effectively suppresses early-stage decomposition up to about 300–330 °C, while having minimal impact on coating rheology and coatability. For scenarios prioritizing delayed high-temperature degradation and enhanced mass retention at intermediate temperatures, 5 wt% h-BN is more suitable. The forward shift of $T_{max}$ and the increased residue at 420 °C suggest improved thermal protection under flame/thermal-shock conditions. Overall, in unaged coatings, well-dispersed low h-BN loadings elevate $T_{onset}$, whereas higher loadings delay $T_{max}$ and increase $W_{\mathrm{res},420}$.

**Figure 11:**
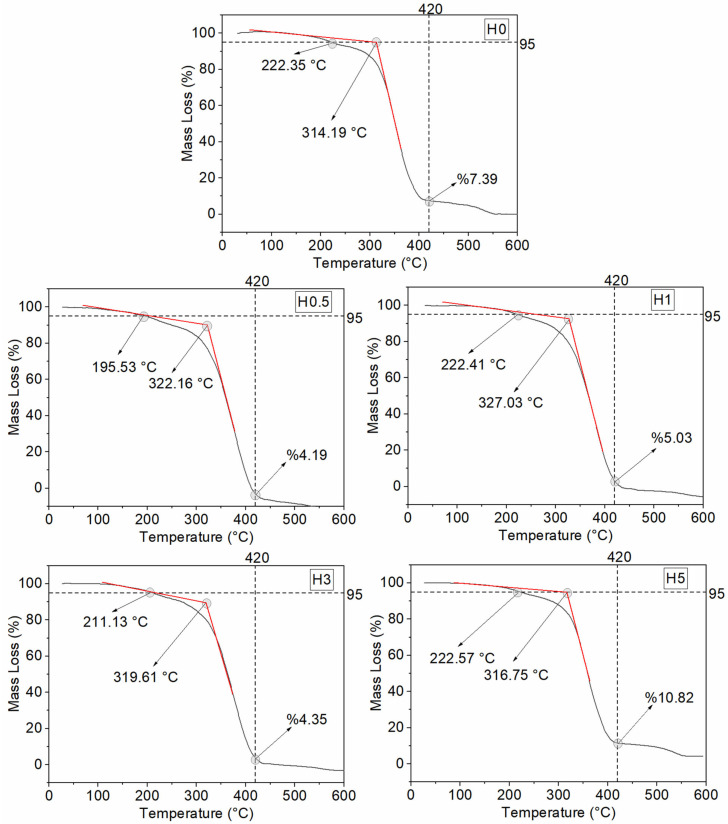
TGA curves of hydrothermally aged coatings.

**Figure 12:**
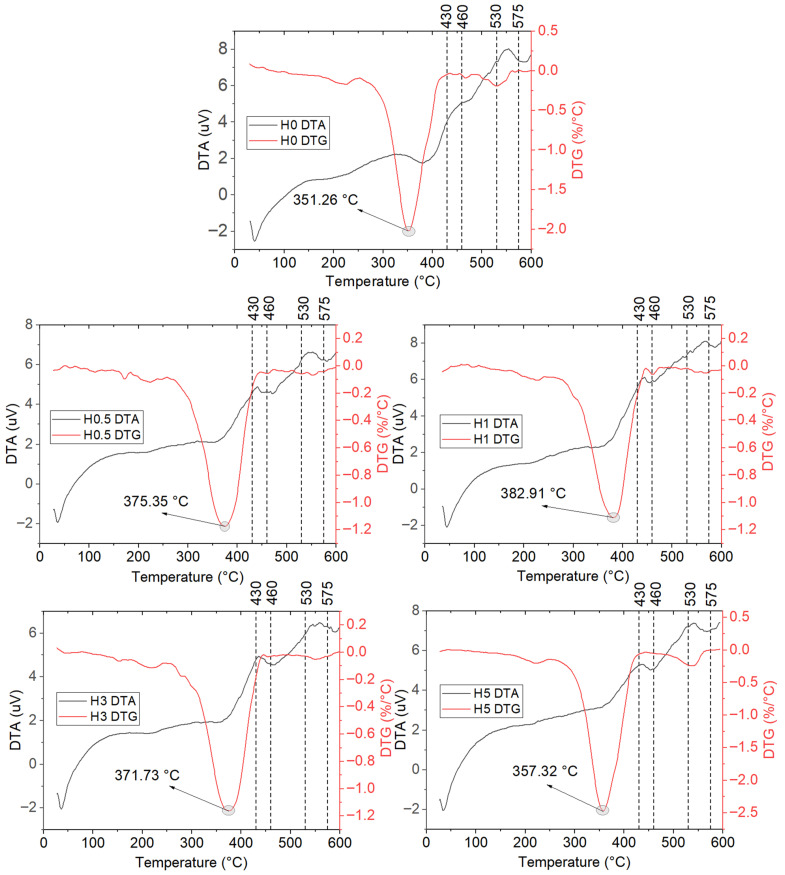
DTA and DTG curves of hydrothermally aged coatings.

The TGA results for the hydrothermally aged coatings are reported in Table 7. The corresponding TGA mass loss curves are presented in Figure 11, and the DTA and DTG curves are shown in Figure 12.

Following hydrothermal aging, the coatings exhibit a clear reduction in the onset of decomposition across the entire series, while the main degradation behavior shows a filler content-dependent, two-sided response. $T_{onset}$ decreases by approximately 5–8 °C for all compositions. This systematic decline is consistent with water-catalyzed hydrolysis of ester linkages in the polyester backbone and water-induced plasticization that increases free volume, thereby triggering chain scission at lower temperatures [50]. Although $T_{5}$ does not follow a uniform trend, a marked drop is observed for H0.5 (217.53 → 195.53 °C), suggesting that at low h-BN levels microvoids at the interface and an insufficient barrier effect facilitate the early escape of volatiles (residual styrene, PDMS cyclics, etc.). By contrast, the slight increases in T_5_ for H1, H3, and H5 can be rationalized by partial wash-out of volatile residues into the medium during aging [51].

The peak temperature of the main degradation exhibits two regimes after aging. In H1, $T_{max}$ increases from 372.56 to 382.91 °C (+10.35 °C), implying that ~1 wt% h-BN best preserves a balanced barrier plus heat-sink network that limits hydrolytic progression and partially stabilizes the matrix. In H0, however, $T_{max}$ drops sharply from 377.53 to 351.26 °C, and in H5 from 383.73 to 357.32 °C (both ≈−26 °C). These sharp decreases indicate, respectively, (i) the vulnerability of the neat matrix to hydrolysis and (ii) defect networks arising from agglomeration at high filler content that ease water–salt ingress along the interface and overshadow the thermal-spreading benefit of h-BN [52]. The small decreases at 0.5 and 3 wt% (≈3–4 °C) suggest that an intermediate barrier is present but interfacial degradation is not fully suppressed.

At 420 °C, the residual mass ($W_{\mathrm{res},420}$) decreases for most compositions (U0.5: 7.07% → H0.5: 4.19%; U1: 7.45% → H1: 5.03%; U3: 6.85% → H3: 4.35%), consistent with an increased yield of low-molecular-weight volatiles via hydrolysis and a reduced char yield at intermediate temperatures [52]. Two exceptions are noteworthy: H0 rises from 4.74% to 7.39%, plausibly due to salt accumulation/inorganic residues retained after aging. H5 rises from 10.39% to 10.82%, attributable to both the higher inorganic filler fraction and salt deposition. The ≈0% residue at 600 °C observed across all coatings mirrors the unaged series and underscores sensitivity to test atmosphere and flow/calibration settings [53].

Comparatively, H1 delivers the most balanced performance under hydrothermal conditions. It retains the highest $T_{onset}$ in the series (327.03 °C) and exhibits an increase in $T_{max}$ after aging, indicating that this composition maintains a favorable balance between chemical stability in saline environment and thermal dissipation. The H5 sample produces the highest residue at 420 °C, yet its sharp decrease in $T_{max}$ reveals a faster overall breakdown of the material. This occurs because elevated filler amounts promote hydrothermal cracking, which effectively cancels out the intended heat-shielding benefits in harsh marine conditions. In practical terms, under moist or marine service, about 1 wt% h-BN provides the best balance between onset and main-stage thermal stability. For flame or thermal-shock conditions, where retaining mass at intermediate temperatures is more critical, 5 wt% h-BN can be advantageous, as long as the associated decrease in $T_{max}$ is considered in the design.

#### 3.2.2. Differential Scanning Calorimetry (DSC) Test Results

**Figure 13:**
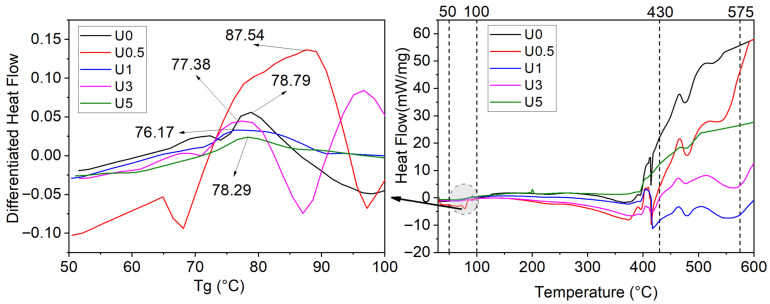
DSC curves of unaged coatings.

**Figure 14:**
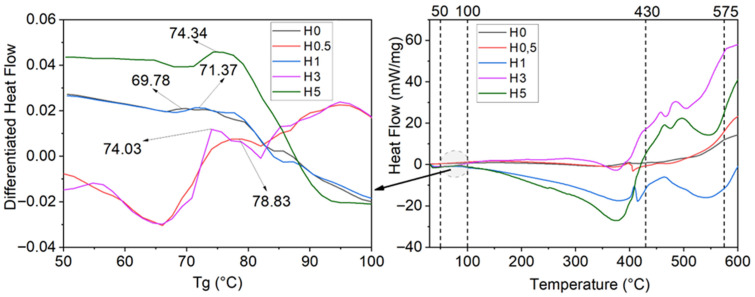
DSC curves of hydrothermally aged coatings.

The DSC results for the unaged and hydrothermally aged coatings (nano-gelcoat) are reported in Table 8, and the DSC corresponding curves are shown in Figure 13 and Figure 14, respectively. In the DSC tables, $T_{g}$ denotes the glass transition temperature.

The $T_{g}$ rises from 78.79 °C in U0 to 87.54 °C in U0.5 (+8.75 °C), then returns toward the neat-resin baseline at higher loadings (U1 = 76.17 °C, U3 = 77.38 °C, U5 = 78.29 °C). This behavior is attributed to well-dispersed, surface-parallel h-BN at about 0.5 wt%. It creates an extended interphase with immobilized polymer chains, reducing free volume and restricting segmental mobility [54]. At ≥1 wt%, agglomeration/void formation reduces the effective interfacial area. Diffusion-limited curing around filler tactoids may slow the MEK-P–cobalt (MEKP–Co) curing kinetics and slightly lower network density. In combination, these effects drive $T_{g}$ back toward (or marginally below) the neat-resin level [55,56].

When read alongside TGA, the trend is consistent, while recognizing the two techniques probe different phenomena ($T_{g}$ reflects segmental mobility; $T_{onset}/T_{max}$ reflect chemical degradation). Notably, $T_{onset}$ increases from U0 → U0.5 → U1 as 319.64 → 328.53 → 334.87 °C, whereas $T_{g}$ peaks at 0.5 wt%. This suggests that barrier/heat-sink effects of h-BN can delay the onset of decomposition, but at 1 wt% curing heterogeneities can depress $T_{g}$ despite the improved $T_{onset}$.

After hydrothermal aging, the $T_{g}$ decreases across the entire series. This systematic reduction can be explained by three main effects. First, hydrolysis causes chain scission in the polyester backbone, lowering the network density. Second, absorbed saline water plasticizes the matrix and increases free volume, and third, micro voiding and swelling at the interfaces enhance segmental mobility [57,58]. The lowest $T_{g}$ in H0 reflects unhindered water diffusion in the absence of a barrier, whereas the fact that H0.5 still exhibits the highest $T_{g}$ after aging indicates that a modest h-BN loading can form lamellar diffusion barriers that limit water uptake and partially immobilize chains at the interphase [59]. At higher loadings (H3/H5), the $T_{g}$ depression becomes more moderate. The ongoing barrier effect is partly offset by agglomeration and debonding, which introduce additional free volume. As a result, $T_{g}$ shows a net, intermediate decrease [60,61]. The more pronounced drop in H1 suggests that, at ~1 wt%, dispersion/interfacial integrity is partly compromised under hydrothermal conditions, allowing localized plasticization.

Taken together with the TGA data, these results show a consistent aging response. Hydrothermal exposure reduces $T_{onset}$ by about 5–8 °C across all groups, and the accompanying drops in $T_{g}$ measured by DSC match expectations based on matrix plasticization and chemical weakening. Notably, $T_{max}$ increases in H1 by ~10 °C, H-BN’s heat-sink and barrier effects still operate at elevated temperatures. Nevertheless, H1’s $T_{g}$ declines by 4.8 °C. This partial “decoupling” between decomposition ($T_{onset}/T_{max}$) and segmental mobility ($T_{g}$) is expected, because $T_{g}$ is governed by free volume and cure state, whereas $T_{onset}/T_{max}$ reflect bond-scission kinetics. Even when high-temperature stability improves, chain mobility at ambient/intermediate temperatures can still rise due to hydrothermal plasticization. In H5, the notable decrease in $T_{max}$ alongside a high 420 °C residue indicates that interfacial defects and enlarged diffusion pathways advance the main degradation event. The elevated inorganic content still preserves intermediate-temperature residue. The moderate $T_{g}$ level observed for H5 is consistent with this trade-off.

For moist/marine service, 0.5 wt% h-BN offers the best balance for segmental stability (higher $T_{g}$). If delaying the main thermal degradation (higher $T_{max}$) is the priority, ~1 wt% may be preferred; however, at this composition, no $T_{g}$ gain should be expected under hydrothermal conditions.

### 3.3. Morphological Analysis

**Figure 15:**
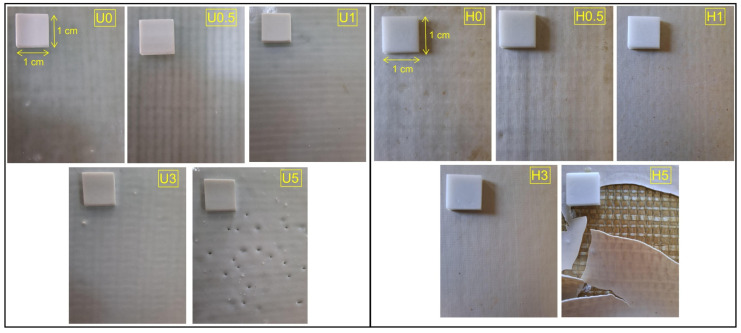
Micro images of unaged (U) and hydrothermally aged (H) coatings.

**Figure 16:**
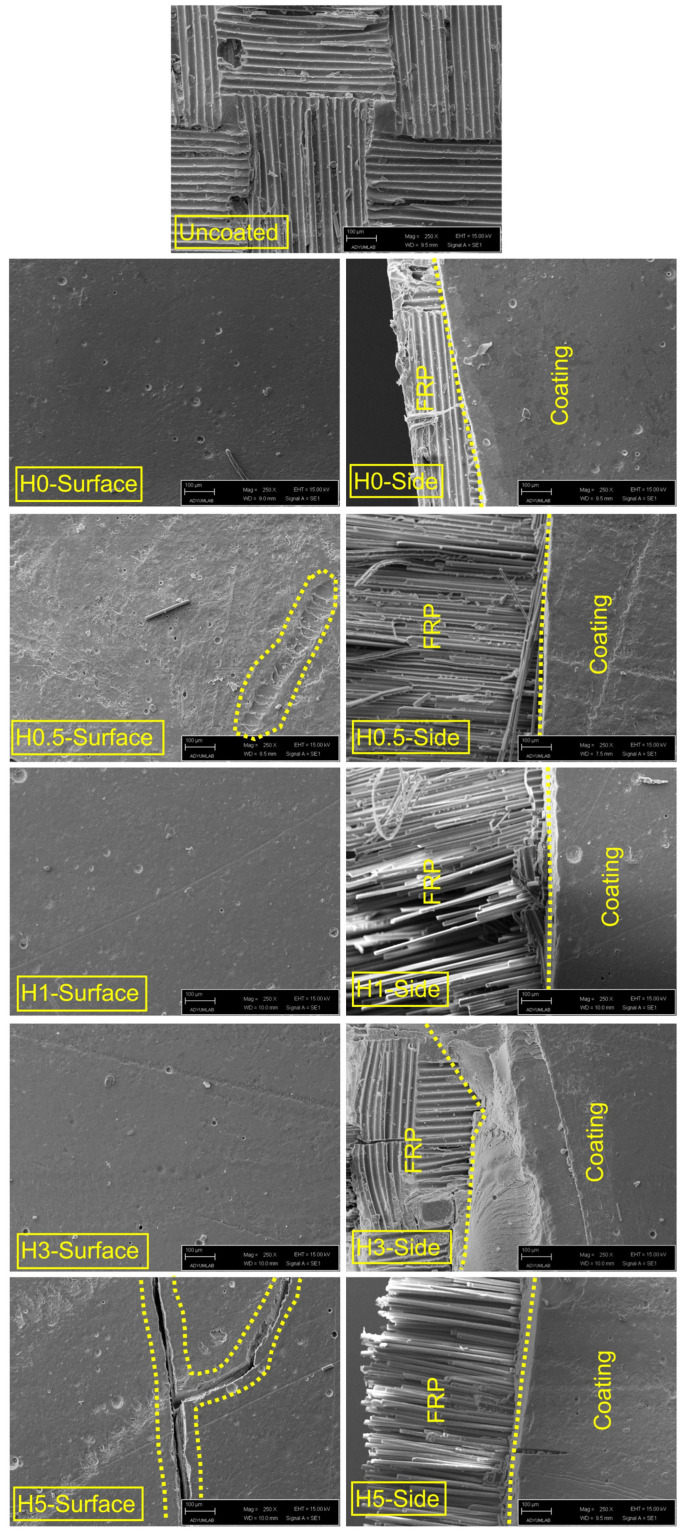
Surface and Side SEM images of hydrothermally aged (H) coatings and FRP composites.

**Figure 17:**
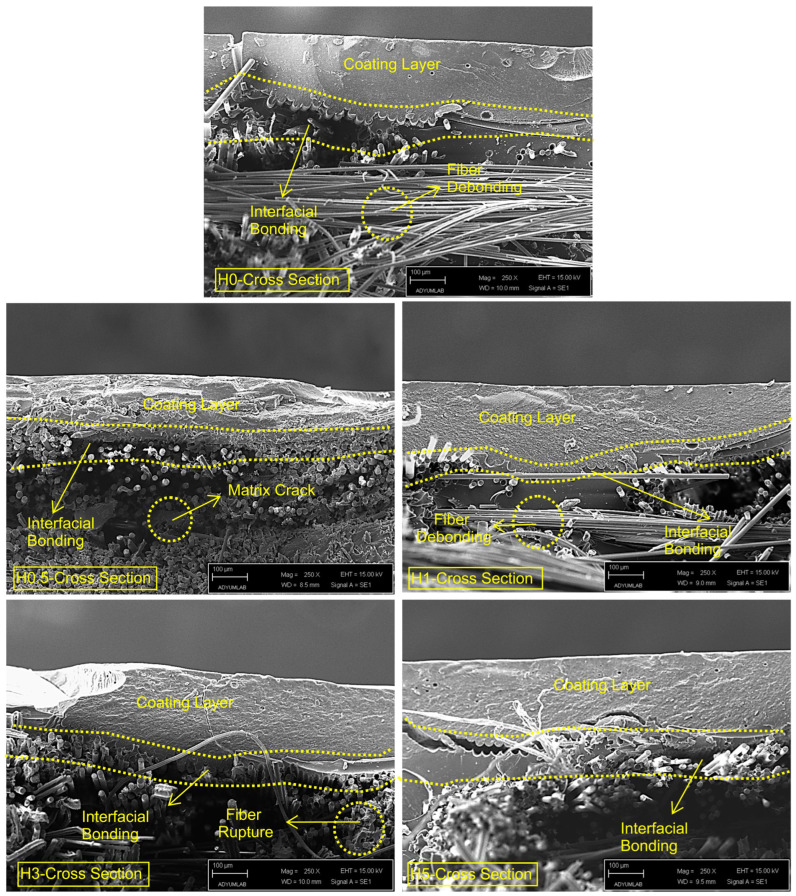
Cross-sectional SEM images of hydrothermally aged (H) coatings and FRP composites.

Surface-view micrographs (1 cm × 1 cm) of the coated FRP plates were taken before and after hydrothermal aging. These images are presented in Figure 15. Post-aging surface and edge SEM micrographs for both uncoated and coated plates are shown in Figure 16, and cross-sectional SEM images are presented in Figure 17. Thickness measurements taken at multiple locations (corroborated by cross-sectional SEM micrographs) indicate an average coating thickness in the range of 400–450 µm.

Figure 15 indicates the presence of micro-voids in the U5 specimen, attributable to h-BN agglomeration. Such defects are not apparent in the other U-series coatings. Following hydrothermal aging, the H5 coatings display pronounced cracking and fragmentation, whereas these features are not observed in the remaining H-series samples. The micro-porosity in the 5 wt% h-BN coatings likely facilitates water ingress, accelerating the aging process and promoting damage in the underlying FRP laminate. The observations are consistent with the diminished mechanical performance recorded for the U5 and H5 series.

Examination of Figure 16 and Figure 17 indicates that the post-aging SEM observations are consistent with the mechanical trends. Relative to the uncoated reference, the tensile strength of the hydrothermally aged, coated specimens decreases to approximately 36–44% of the baseline across H0 to H5, while the flexural strength falls to about 40–50%. By contrast, the elastic modulus is retained at roughly 63–72% and the flexural modulus at about 70–90%, with the latter depending on h-BN content. The same trend was observed in creep and stress relaxation test results. Collectively, these findings indicate that seawater absorption and thermal-osmotic cycling cause plasticization, partial hydrolysis, and void formation within the polyester matrix and at the interface. Consequently, this loss of structural continuity hinders load transfer, leading to failure primarily through interfacial cracks and fiber debonding.

In H0, surface micrographs reveal widespread micro-voiding with localized blistering/flaking, while the cross-section and edge views show a discontinuous band with fine gaps along the coating–composite boundary. Together with fiber bundle slip traces aligned with bundle channels, this morphology rationalizes the pronounced losses in strength (tensile and flexural reduced to roughly 40–43% of the reference). The absence of a nanoparticle barrier in the h-BN-free coating is consistent with short diffusion pathways, weakened interfacial adhesion, and degraded load transfer.

In H0.5, the surface exhibits longitudinal microcracks and a more orderly yet still dense pit (micro-void) topography. This partial improvement in integrity aligns with the relatively high retention of flexural stiffness (≈82% of the reference). However, because interfacial discontinuities persist, tensile and flexural strengths remain close to those of H0. A modest h-BN fraction, aided by the hydrophobic contribution of PDMS, slows diffusion but does not yet produce a continuous, well-bonded interphase [62].

In H1, the surface is more homogeneous and topographically smooth, and the cross-section shows a more continuous line of contact at the coating–laminate boundary. Fiber debonding is limited, and matrix microcracks are shorter and more dispersed. The mechanical maxima in this series (tensile strength 358.75 MPa, flexural strength 531.29 MPa, and notably ≈90% retention of flexural modulus) are consistent with this improved microstructural integrity. At this loading, well-dispersed h-BN provides an effective barrier, promotes crack deflection/bridging and stress redistribution, and sustains an interphase capable of efficient load transfer.

In H3, the surface shows fewer but longer microcracks and pit clusters that tend to coalesce; the edge regions display wedge-shaped separation zones and local void clusters. Partial fiber breaks and bundle-end openings indicate rising interfacial stress concentrations [63]. This damage motif matches the renewed decline in strength relative to H1 and the pullback of stiffness metrics to ≈71–83% of the reference, pointing to the onset of agglomeration and rheology/wetting limitations.

In H5, Y-shaped microcrack networks and discontinuous surface grooves are evident, with pervasive interfacial voids in cross-section, extensive fiber pull-out, and an irregular coating–composite boundary. The high h-BN content increases viscosity and fosters particle clustering, which weakens wetting and amplifies initial defects and modulus mismatch [64,65]. These features explain the lowest tensile/flexural strengths in the series and flexural modulus retention falling to about 70%. Agglomeration-centered stress concentrations facilitate crack initiation and guide crack advance along the interface.

Taken together, the H-series indicates that interfacial integrity loss is the dominant hydrothermal damage mechanism: pits/blisters, microcracks, and voids preferentially channel to the fiber–matrix boundary, promoting debonding, fiber pull-out, and interrupted load paths. Maintaining a low h-BN concentration of about 1 wt% best protects the coating because it effectively blocks saltwater and deflects cracking. In contrast, adding too much filler creates flow and wetting problems that expand existing micro-defects. Consequently, H1 delivers the closest approach to an optimal morphology–mechanical balance.

### 3.4. Multi-Criteria Decision-Making (MCDM) Results

**Figure 18:**
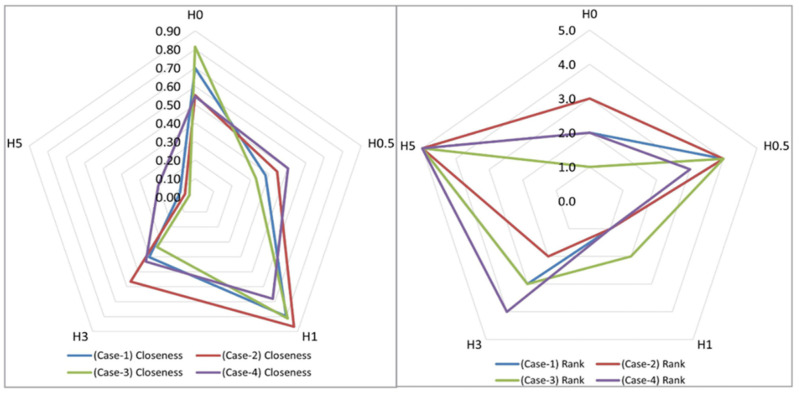
Radar charts of closeness and rank.

The MCDM TOPSIS evaluation was performed for five hydrothermally aged samples (H0, H0.5, H1, H3, H5); the uncoated laminate served only as an external baseline and was not ranked. The results were shown in Table 9. The radar chart of closeness and rank was shown in Figure 18.

Across all weighting scenarios, the results are consistent with the single-criterion trends discussed in the manuscript: the 1 wt% formulation (H1) generally provides the most balanced response, whereas H5 persistently underperforms due to multi-domain penalties.

In Case-1 (Equal weights), uniform weighting across the eleven criteria favors H1, with H0 as the closest follower and H3/H0.5 forming the middle tier. H5 ranks last. In Case-2 (mechanical 60%, viscoelastic 20%, thermal 20%), when mechanical metrics dominate, H1 strengthens its lead; H3 typically places second, followed by H0/H0.5, and H5 remains last. This pattern reflects the superior strengths and modulus retained by H1 under aging. In Case-3 (viscoelastic 60%, mechanical 20%, thermal 20%), under viscoelastic emphasis, H0 and H1 are essentially neck-and-neck; H0 attains a slightly higher closeness coefficient C than H1, but the difference is marginal (i.e., within rounding at the third decimal). The near-tie indicates that small changes in ΔJ, $\Delta E_{c}$, and quasi-steady creep rate (after normalization) can offset the mechanical advantage of H1. In Case-4 (Thermal 60%, mechanical 20%, viscoelastic 20%), in the thermal-emphasized scenario, H0 again exhibits the highest C, with H1 extremely close behind (the gap is small enough to be practically negligible at typical reporting precision). This outcome is plausible given that (i) the three thermal indicators are weighted more heavily as a group, and (ii) the residual 40% share (mechanical + viscoelastic) may not be sufficient to overcome the small, thermally driven advantage of H0 once values are normalized to column spreads. In other words, under a strongly thermal-centric view, H0 and H1 behave as statistical near-ties.

When the four scenarios are combined using both average C and average rank, they give a consistent overall order: H1 > H0 > H3 > H0.5 > H5. Importantly, H1 always remains the best option and H5 the worst, even when the criterion weights are varied within a reasonable range, showing that the multi-criteria result is reliable. The small reversals between H0 and H1 in Case-3 and Case-4 are caused by two effects. First, benefit/cost normalization interacts with the very small differences between alternatives in some criteria, and second, the category weights are redistributed. These effects do not change the main conclusion that H1 is still the most balanced choice across the mechanical, viscoelastic, and thermal domains.

### 3.5. PDMS–h-BN Synergistic Protection Mechanism

The improved hydrothermal resistance of the h-BN/PDMS nano-gelcoat can be attributed to a complementary dual-scale protection mechanism. At the surface scale, PDMS migrates toward the coating–air interface during curing because of its low surface energy (~20 mJ/m^2^). This migration leads to the formation of a hydrophobic layer. As a result, the thermodynamic driving force for moisture adsorption decreases, and the initial ingress of water is limited [27]. At the bulk scale, well-dispersed h-BN platelets form a tortuous diffusion network. This network substantially increases the effective diffusion path length of moisture through the coating thickness [41]. In addition to these separate effects, PDMS also serves as a processing aid. Its low surface tension reduces van der Waals attraction between platelets during ultrasonication. This improves h-BN dispersion quality and helps preserve a high effective platelet aspect ratio, which is important for blocking diffusing water molecules. This processing synergy appears to reach an optimum at approximately 1 wt% h-BN. At this level, the platelet concentration is high enough to create a percolating barrier network, while agglomeration is still avoided. The consistent superiority of H1 over the PDMS-only control (H0) in all test categories provides indirect experimental evidence for this synergistic mechanism. SEM observations revealed a more continuous coating–laminate interface. Direct verification of this mechanism should be considered a priority in future studies.

## 4. Conclusions

This study demonstrated that polyester-based nano-gelcoats reinforced with hexagonal boron nitride (h-BN) nanoparticles can significantly improve the hydrothermal durability of fiber-reinforced polymer (FRP) composite coatings for marine applications. Through a combination of mechanical, viscoelastic, thermal, and morphological evaluations, the 1 wt% h-BN loading was identified as the optimal formulation.

After 90 days of aging in seawater at 80 °C, the 1 wt% h-BN nano-gelcoat retained about 44% of tensile strength and 90% of flexural stiffness, better than both the uncoated and higher-loaded versions. It also showed the least creep (~4638 µε) and highest stress retention (~82.2%), making it the most durable option under long-term loading.

Thermal tests showed that the 1 wt% h-BN nano-gelcoat had a peak decomposition temperature ($T_{max}$) of ~383 °C and its glass transition temperature ($T_{g}$) decreased by only ~4.8 °C after aging. In contrast, the h-BN-free system showed a much larger $T_{max}$ drop of ~26 °C and a $T_{g}$ decrease of ~9 °C. Morphological observations supported these trends. 1 wt% nano-gelcoat maintained a smooth, crack-free surface with strong interfacial adhesion, whereas higher filler loadings resulted in agglomeration, interfacial voids, and microcracking that impaired overall performance.

A TOPSIS analysis using eleven criteria confirmed that the 1 wt% h-BN coating (H1) consistently ranked best across all weighting scenarios, with the highest closeness score and lowest average rank. The final order was H1 > H0 > H3 > H0.5 > H5, showing that a low h-BN loading (~1 wt%) offers the best balance of mechanical, viscoelastic, and thermal performance after aging, while the 5 wt% loading performed worst.

These findings confirm that low h-BN loadings, especially around 1 wt%, provide a balanced performance. They improve barrier function, thermal conductivity, and interfacial integrity at the same time. From an industrial perspective, the 1 wt% h-BN/PDMS-modified polyester nano-gelcoat shows strong potential as a practical protective layer for marine FRP structures. It may help improve durability and reduce life-cycle maintenance demands under severe hydrothermal service conditions. Future work may explore hybrid filler systems and long-term field validation to further enhance protective functionality in real-world marine environments.

## Figures and Tables

**Table 1:** Mechanical test standards.

Tests	Standards	Sizes (mm)	Span Length	Speed (mm/min)
Tensile Test	ASTM D3039 [32]	250 × 25 × 2.5	---	2
Bending Test	ASTM D790 [33]	127 × 12.7 × 2.5	80	1
Creep Test	ASTM 2990 [34]	127 × 12 × 2.5	---	1
Stress Relaxation Test	ASTM E328 [35]	250 × 25 × 2.5	---	0.48

**Table 2:** Multi-criteria decision-making parameters.

Criterion	Abbrev.	Type	Category	Case 1	Case 2	Case 3	Case 4
Tensile Strength	TS	Benefit	Mechanical	8.33	15	5	5
Tensile Modulus	TM	Benefit	Mechanical	8.33	15	5	5
Flexural Strength	FS	Benefit	Mechanical	8.33	15	5	5
Flexural Modulus	FM	Benefit	Mechanical	8.33	15	5	5
QS Creep Rate	QCP	Cost	Viscoelastic	8.33	5	15	5
ΔJ	ΔJ	Cost	Viscoelastic	8.33	5	15	5
ΔE_c_	ΔE_c_	Cost	Viscoelastic	8.33	5	15	5
Stress Retention	SR	Benefit	Viscoelastic	8.33	5	15	5
T_onset_	T_onset_	Benefit	Thermal	11.11	6.7	6.7	20
T_max_	T_max_	Benefit	Thermal	11.11	6.7	6.7	20
T_g_	T_g_	Benefit	Thermal	11.11	6.7	6.7	20

**Table 3:** Tensile and flexural test results.

	Tensile Strength (MPa)	Percentage Loss of TS (%)	Modulus of Elasticity (GPa)	Percentage Loss of MOE (%)	Flexural Strength (MPa)	Percentage Loss of FS (%)	Flexural Modulus (GPa)	Percentage Loss of FM (%)
Uncoated	822.04 ± 24.7		46.09		1070.52 ± 34.3		38.67	
H0	332.08 ± 11.6	59.6	31.90	30.8	464.02 ± 17.2	56.7	29.33	24.2
H0.5	333.26 ± 13.3	59.5	30.22	34.4	466.62 ± 16.3	56.4	31.71	18.0
H1	358.75 ± 14.7	56.4	33.22	27.9	531.29 ± 21.2	50.4	34.81	10.0
H3	347.37 ± 12.5	57.7	32.78	28.9	497.15 ± 18.9	53.6	32.26	16.6
H5	298.77 ± 9.6	63.7	29.07	36.9	430.19 ± 15.5	59.8	26.91	30.4

**Table 4:** Creep test results.

	Quasi-Steady Creep Rate	Creep Compliance	Increase in Creep Strain	Creep Modulus	Creep Modulus	Change in Creep Modulus
	έ(t) (s^−1^)	ΔJ (%)	Δε (µε)	E_cp_(t) (GPa)	E_c3h_(t) (GPa)	ΔE_c_ (%)
Uncoated	3.90333 × 10^−7^	10.18	4784.72	6.61	6.00	9.28
H0	4.91826 × 10^−7^	13.78	4804.04	5.26	4.55	13.46
H0.5	4.99803 × 10^−7^	23.83	3653.97	8.69	7.00	19.38
H1	4.12844 × 10^−7^	17.45	4637.54	4.41	3.84	13.08
H3	6.05705 × 10^−7^	22.18	4662.32	7.38	6.04	18.15
H5	6.19735 × 10^−7^	26.98	4687.72	6.86	5.40	21.25

**Table 5:** Stress relaxation test results.

	Stress Retention (%)	E_rp_(t) (GPa)	E_r3h_(t) (GPa)	Relaxation Ratio
Uncoated	83.31	36.05	30.04	0.83
H0	81.49	31.97	26.05	0.81
H0.5	78.97	32.40	25.58	0.79
H1	82.21	29.23	24.03	0.82
H3	79.57	31.55	25.10	0.80
H5	77.08	29.78	22.96	0.77

**Table 6:** TGA test results of unaged coatings.

	T_5_ (°C)	T_onset_ (°C)	T_max_ (°C)	W_res,420_ (%)	W_res,600_ (%)
U0	222.61	319.64	377.53	4.74	≈0
U0.5	217.53	328.53	379.11	7.07	≈0
U1	219.68	334.87	372.56	7.45	≈0
U3	205.99	327.53	374.92	6.85	≈0
U5	221.04	324.48	383.73	10.39	≈0

**Table 7:** TGA test results of hydrothermally aged coatings.

	T_5_ (°C)	T_onset_ (°C)	T_max_ (°C)	W_res,420_ (%)	W_res,600_ (%)
H0	222.35	314.19	351.26	7.39	≈0
H0.5	195.53	322.16	375.35	4.19	≈0
H1	222.41	327.03	382.91	5.03	≈0
H3	211.13	319.61	371.73	4.35	≈0
H5	222.57	316.75	357.32	10.82	≈0

**Table 8:** DSC test results of unaged and hydrothermally aged coatings.

	T_g_ (°C)		T_g_ (°C)
U0	78.79	H0	69.78
U0.5	87.54	H0.5	78.83
U1	76.17	H1	71.37
U3	77.38	H3	74.03
U5	78.29	H5	74.34

**Table 9:** The closeness and rank results.

	(Case-1)		(Case-2)		(Case-3)		(Case-4)		Avg. Closeness	Avg. Rank
	Closeness	Rank	Closeness	Rank	Closeness	Rank	Closeness	Rank		
H0	0.699	2	0.552	3	0.814	1	0.546	2	0.653	2
H0.5	0.381	4	0.445	4	0.330	4	0.503	3	0.415	3.75
H1	0.799	1	0.867	1	0.813	2	0.682	1	0.790	1.25
H3	0.403	3	0.566	2	0.334	3	0.432	4	0.434	3
H5	0.082	5	0.055	5	0.031	5	0.200	5	0.092	5

## Data Availability

The data presented in this study is available on request from the corresponding author.
